# A nuclear shift of GSK3β protein is an independent prognostic factor in prostate cancer

**DOI:** 10.18632/oncotarget.26739

**Published:** 2019-03-01

**Authors:** Till Eichenauer, Mohammad Hussein, Claudia Hube-Magg, Martina Kluth, Franziska Büscheck, Doris Höflmayer, Maria Christina Tsourlakis, Stefan Steurer, Till S. Clauditz, Andreas M. Luebke, Eike Burandt, Waldemar Wilczak, Andrea Hinsch, David Dum, Burkhard Beyer, Thomas Steuber, Hartwig Huland, Markus Graefen, Ronald Simon, Guido Sauter, Nathaniel Melling, Thorsten Schlomm, Sarah Minner

**Affiliations:** ^1^ Institute of Pathology, University Medical Center Hamburg-Eppendorf, Hamburg, Germany; ^2^ Department of Urology, University Medical Center, Hamburg-Eppendorf, Hamburg, Germany; ^3^ Martini-Clinic, Prostate Cancer Center, University Medical Center Hamburg-Eppendorf, Hamburg, Germany; ^4^ Department of General, Visceral and Thoracic Surgery, University Medical Center Hamburg-Eppendorf, Hamburg, Germany; ^5^ Department of Urology, Charité-Universitätsmedizin Berlin, Berlin, Germany

**Keywords:** GSK3beta, prostate cancer, prognosis, immunohistochemistry

## Abstract

Glycogen synthase kinase 3ß (GSK3ß) regulates many cancer relevant cellular processes and represents a potential therapeutic target. GSK3ß overexpression has been linked to adverse tumor features in many cancers, but its role in prostate cancer remains uncertain. We employed immunohistochemical GSK3ß expression analysis on a tissue microarray with 12,427 prostate cancers. Cytoplasmic and nuclear GSK3ß staining was separately analyzed. GSK3ß staining was absent in normal prostate epithelium, whereas 57% of 9,164 interpretable cancers showed detectable GSK3ß expression. Cytoplasmic staining was considered weak, moderate, and strong in 36%, 19.5% and 1.5% of tumors and was accompanied by nuclear GSK3ß staining in 47% of cases. Cytoplasmic GSK3ß staining as well as nuclear GSK3ß accumulation was associated with advanced tumor stage, high Gleason grade, presence of lymph node metastasis and early biochemical recurrence (*p* < 0.0001 each for cytoplasmic staining and nu-clear accumulation). Prognosis of GSK3ß positive cancers became particularly poor if nuclear GSK3ß staining was also seen (*p* < 0.0001). The prognostic impact of nuclear GSK3ß accumu-lation was independent of established preoperative and postoperative parameters in multivari-ate analyses (*p* < 0.0001). The significant association of GSK3ß expression with deletions of *PTEN*, 3p13 (*p* < 0.0001 each), 5q21 (*p* = 0.0014) and 6q15 (*p* = 0.0026) suggest a role of GSK3ß in the development of genomic instability. In summary, the results of our study identify GSK3ß as an independent prognostic marker in prostate cancer.

## INTRODUCTION

Prostate cancer is the 2nd most prevalent cancer in men in Western societies [1], but only a small subset is highly aggressive and requires extensive treatment [2, 3]. Presently Gleason grade, tumor extent on biopsies, prostate-specific antigen (PSA), and clinical stage are recognized prognostic parameters. These factors are statistically powerful, but not always sufficient for individual treatment decisions. Thus it is hoped that new biomarkers will enable a more reliable prediction of prostate cancer aggressiveness.

Glycogen synthase kinase 3ß (GSK3ß) is a ubiquitously expressed multifunctional serine/threonine kinase that was originally named after its function as an enzyme in glycogen biosynthesis. It also plays a key role in regulating a multitude of other pathways affecting metabolism, proliferation, survival and cell motility [4]. GSK3ß shuttles between the cytoplasm and the nucleus where it is believed to exert distinct functions [5]. Deregulation of GSK3ß has been implicated in the development of many human diseases, including diabetes, cardiovascular diseases, Alzheimer’s, Parkinson’s, and cancer [4]. Overexpression of GSK3ß has been linked to adverse tumor phenotype and poor prognosis in several cancer types, including breast [6, 7], ovarian [8], oral cavity [9], urinary bladder [10], non-small cell lung [11], gastric [12], and pancreatic cancers [13]. Based on these findings, GSK3ß has gained considerable interest as a target for novel therapies. At present, more than 50 GSK3ß inhibitors have been described [4] and clinical phase 1/2 trails have been initiated in pancreatic cancer (NCT01632306) and leukemia (NCT01214603). Accumulating evidence suggests that GSK3ß may also be clinically relevant in prostate cancer [14, 15]. Here, GSK3ß is known to be involved in the regulation of androgen receptor (AR) stability, localization, and androgen-stimulated gene expression [16–22]. Two studies analyzing GSK3ß expression on clinical samples from 79 and 499 prostate cancer patients suggested associations between GSK3ß overexpression and high Gleason score [22] and potentially also poor patient prognosis [15].

To study the impact of GSK3ß expression on prostate cancer phenotype and patient prognosis, we analyzed cytoplasmic and nuclear GSK3ß expression in more than 12,400 prostate cancer specimens using a preexisting tissue microarray (TMA).

## RESULTS

### Technical issues

A total of 9,164 of 12,427 tumor samples (74%) were interpretable in our TMA analysis. Reasons for non-informative cases (*n* = 3,263; 26%) included lack of tissue samples or absence of unequivocal cancer tissue in the TMA spot.

### GSK3ß expression in normal and cancerous prostate tissues

**Figure 1:**
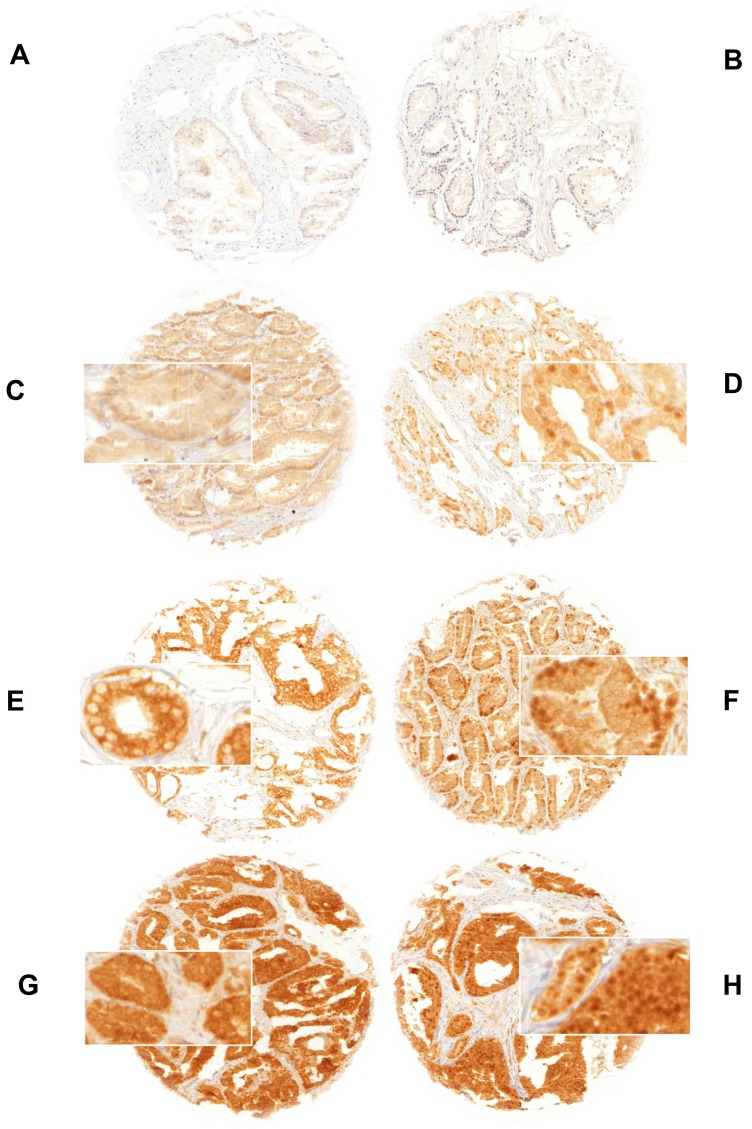
GSK3ß staining of (**A**) negative normal prostate tissue, (**B**) negative prostate cancer, (**C**) weak cytoplasmic only (**D**) weak cytoplasmic and nuclear accumulation, (**E**) moderate cytoplasmic only (**F**) moderate cytoplasmic and nuclear accumulation, (**G**) strong cytoplasmic only and (**H**) strong cytoplasmic and nuclear accumulation. Spot size is 0.6 mm at 100× (inset 400×) magnification. Nuclear accumulation denotes nuclear staining with/without cytoplasmic staining.

**Figure 2:**
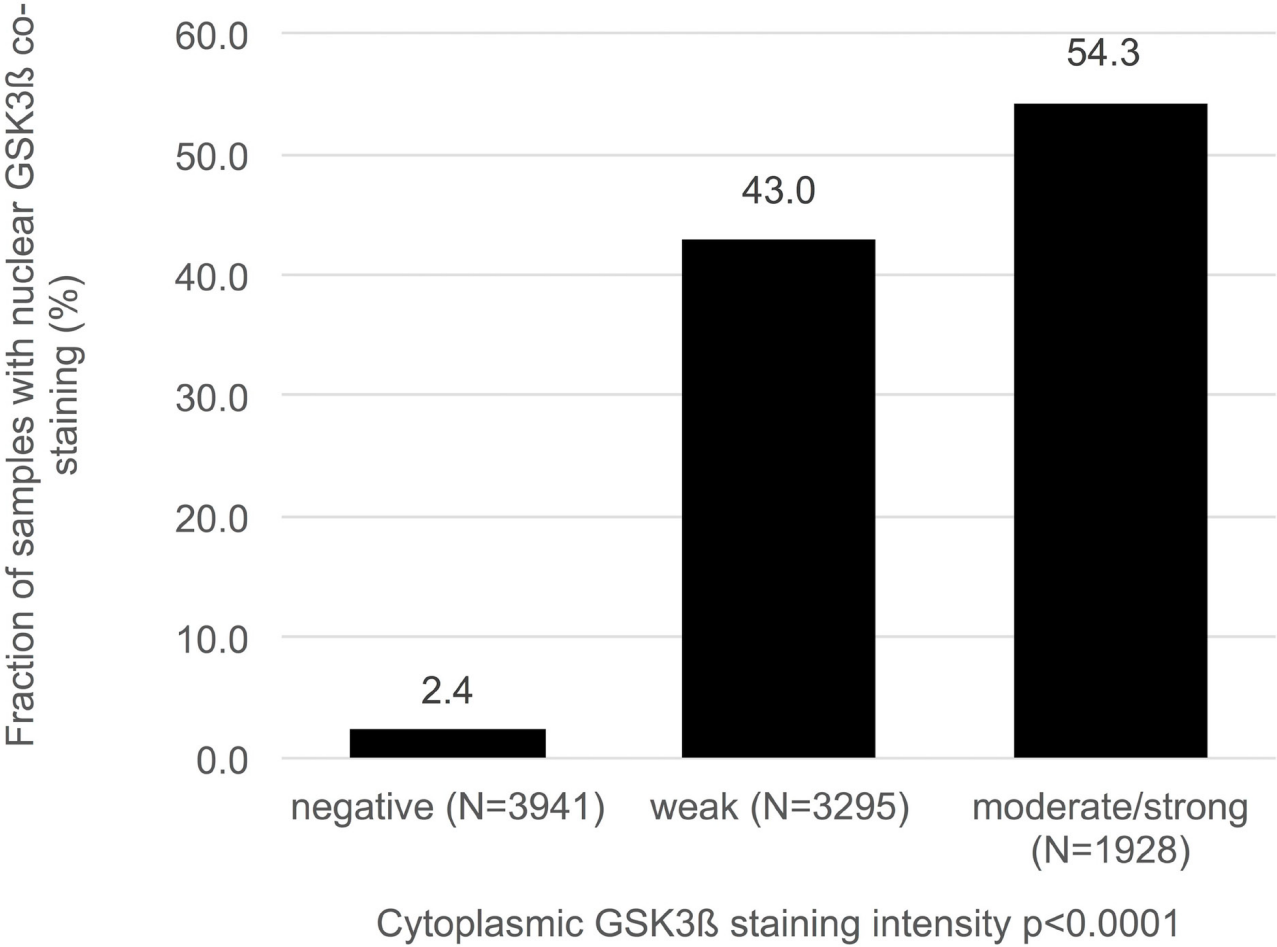
Association between cytoplasmic and nuclear GSK3ß staining

Normal prostate tissue was negative for GSK3ß. In cancers, GSK3ß staining was localized in the cytoplasm and/or in the nucleus. Representative images of cytoplasmic and nuclear GSK3ß staining are given in Figure 1. Cytoplasmic staining (irrespective of nuclear staining) was seen in 5,223 of our 9,164 (57%) interpretable prostate cancers and was considered weak in 36%, moderate in 19.5% and strong in 1.5% of cases. Cytoplasmic and nuclear staining was tightly linked: Cytoplasmic staining was accompanied by nuclear staining in 2,465 (47%) of 5,223 cases and the likelihood for nuclear tumor cell staining rose with increasing levels of cytoplasmic staining (Figure 2; *p* < 0.0001). Nuclear staining without cytoplasmic staining was seen in only 95 cases (1%). To better understand the individual impact of cytoplasmic and nuclear staining, we re-grouped all cancers for the subsequent analyses according to the following criteria: no staining at all (negative, *n* = 3,846), cytoplasmic staining without nuclear co-staining (cytoplasmic only, *n* = 2,758), and cytoplasmic staining with nuclear co-staining (nuclear accumulation, *n* = 2,560, including the 95 cancers with isolated nuclear staining).

### Association with androgen receptor (AR)

**Figure 3:**
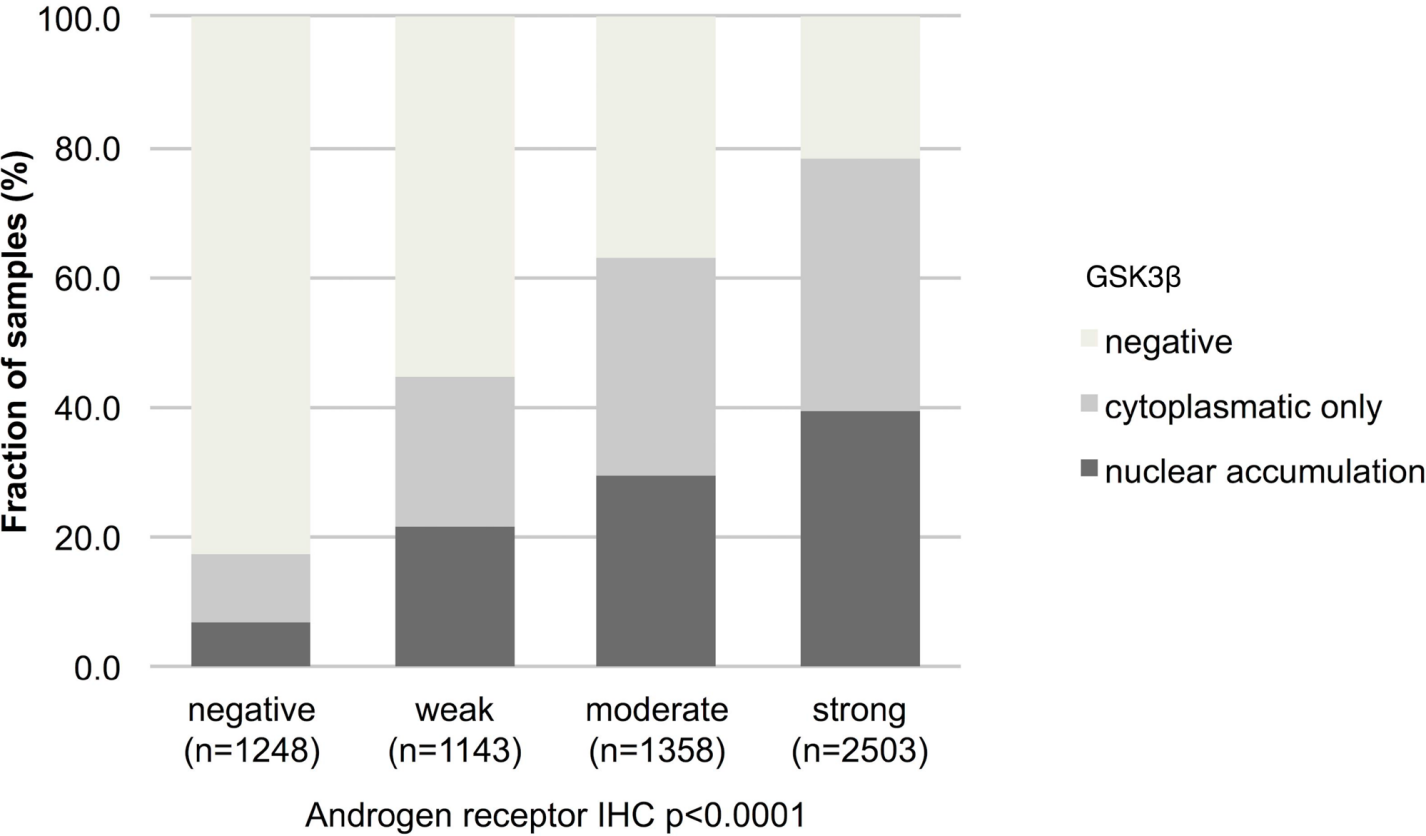
Association between GSK3ß staining pattern and expression of androgen receptor Nuclear accumulation denotes nuclear staining with/without cytoplasmic staining.

As GSK3ß is an AR regulated gene, we compared data on AR expression from a previous study [23] with GSK3ß expression patterns. IHC data on both GSK3ß and AR were available from 6,253 cancers. As expected, there was a strong positive association between AR expression and presence of both cytoplasmic and nuclear GSK3ß protein (*p* < 0.0001 each; Figure 3). Also nuclear GSK3β and nuclear AR expression correlated as well (Supplementary Figure 1).

### Association with *TMPRSS2:ERG* fusion status and ERG protein expression

**Figure 4:**
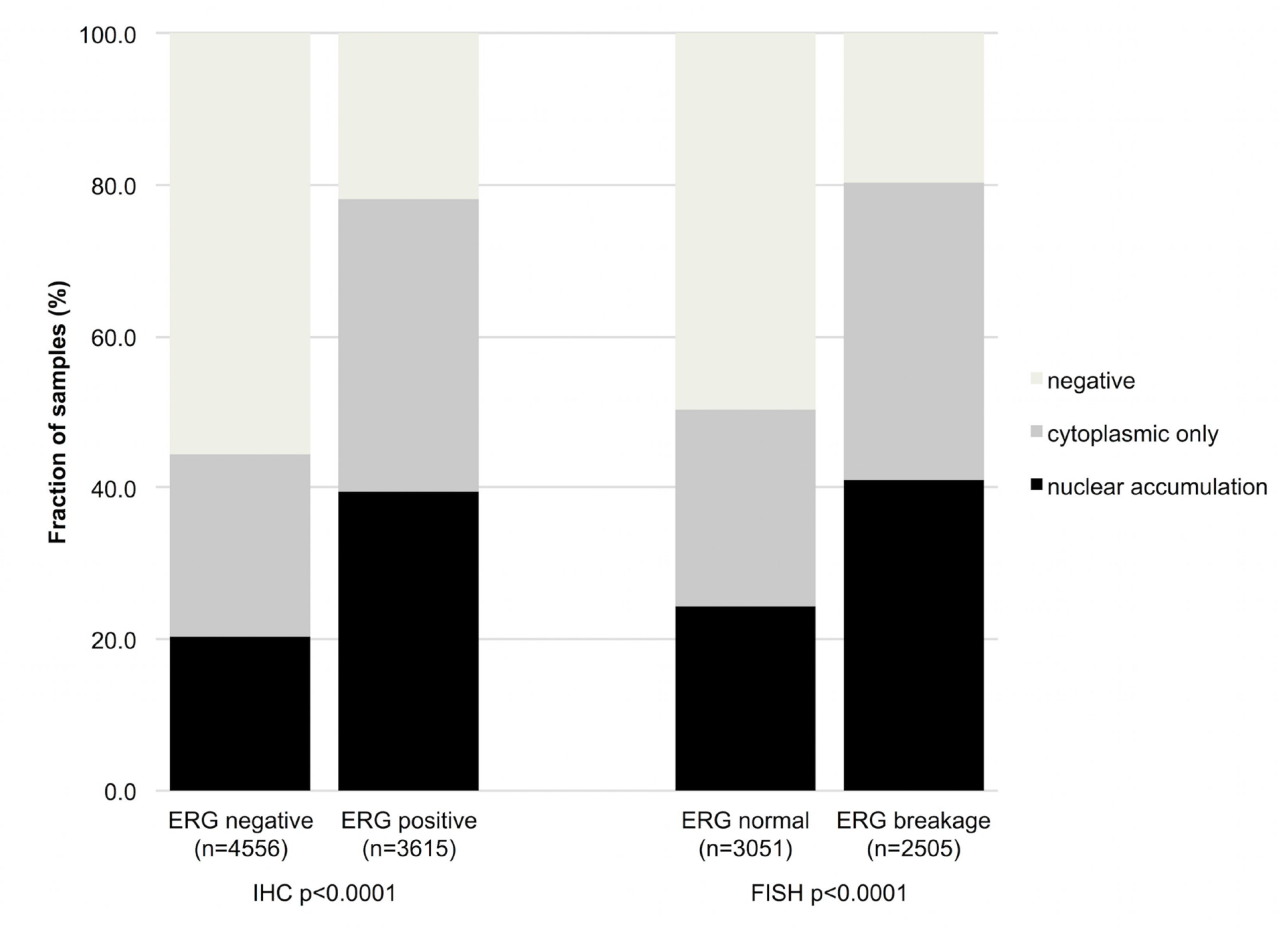
Association between increasing GSK3ß staining and ERG status determined by IHC and FISH Breakage indicates rearrangement of the *ERG* gene by FISH.

Data on *TMPRSS2:ERG* fusion status obtained by FISH were available from 5,556 and by IHC from 8,171 tumors with evaluable GSK3ß staining. Data on both ERG FISH and IHC were available from 5,365 of these cancers, and an identical result (ERG IHC positive and break by FISH or ERG IHC negative and missing break by FISH) was found in 5,137 of 5,365 (95.8%) cancers. Both cytoplasmic expression and nuclear accumulation GSK3ß were strongly linked to *TMPRSS2:ERG* rearrangement and ERG expression (*p* < 0,0001 each, Figure 4). For example, GSK3ß staining was seen in 44.5% of ERG-IHC negative but in 78.3% of ERG-IHC positive cancers.

### Associations with tumor phenotype

Both the intensity of cytoplasmic GSK3ß staining and the presence of nuclear GSK3ß accumulation showed significant associations with adverse tumor features. This was particularly true for nuclear GSK3ß accumulation, which was associated with advanced tumor stage (*p* < 0.0001), high Gleason grade (*p* < 0.0001), lymph node metastasis (*p* < 0.0001), positive surgical margin (*p* < 0.0001) and high preoperative PSA level (*p* = 0.0002, Table 1). Cytoplasmic GSK3ß expression levels showed comparable but somewhat weaker associations (Table 1). All these associations held true in the subset of ERG negative and ERG positive cancers (Supplementary Tables 1 and 2).

**Table 1:** Association between cytoplasmic and nuclear GSK3ß immunostaining and prostate cancer phenotype

		Cytoplasmic GSK3ß (%)					Cytoplasmic and nuclear GSK3ß (%)		
Parameter	N	Negative	Weak	Moderate	Strong	P	Cytoplasmic only	Nuclear accumulation	P
**All cancers**	9,164	43.0	36.0	19.5	1.5		29.6	27.4	
**Tumor stage**									
pT2	5,596	46.8	35.1	16.9	1.2	< 0.0001	30.4	22.8	< 0.0001
pT3a	2,121	36.7	37.9	23.4	2.0		29.0	34.3	
pT3b-pT4	1,140	34.9	36.9	25.8	2.5		25.4	39.7	
**Gleason grade**									
≤3+3	1,922	58.3	32.8	8.8	0.2	< 0.0001	27.7	14.0	< 0.0001
3+4	5,030	41.9	36.7	19.9	1.6		32.1	26.0	
3+4 Tert.5	337	38.3	39.8	20.2	1.8		27.7	34.0	
4+3	903	30.8	40.1	26.0	3.1		30.1	39.1	
4+3 Tert.5	500	29.4	33.0	34.8	2.8		22.5	48.1	
≥4+4	466	34.6	33.3	29.6	2.6		21.3	44.1	
**Lymph node metastasis**									
N0	5,250	40.1	36.9	21.0	2.0	< 0.0001	29.6	30.3	< 0.0001
N+	524	32.8	34.7	30.0	2.5		24.2	43.0	
**Preoperative PSA level (ng/ml)**									
< 4	1,319	41.8	37.9	18.7	1.6	0.0731	33.1	25.2	< 0.0001
4-10	5,319	42.4	36.2	19.8	1.7		30.1	27.5	
10-20	1,827	45.1	34.7	19.2	1.0		27.1	27.8	
>20	653	45.2	33.4	19.9	1.5		21.2	33.7	
**Surgical margin**									
Negative	7,253	43.8	36.3	18.4	1.5	< 0.0001	30.1	26.1	< 0.0001
Positive	1,802	40.3	34.4	23.4	1.9		27.0	32.7	

Nuclear accumulation: nuclear staining with or without cytoplasmic co-staining.

### Association to other key genomic deletions

**Figure 5:**
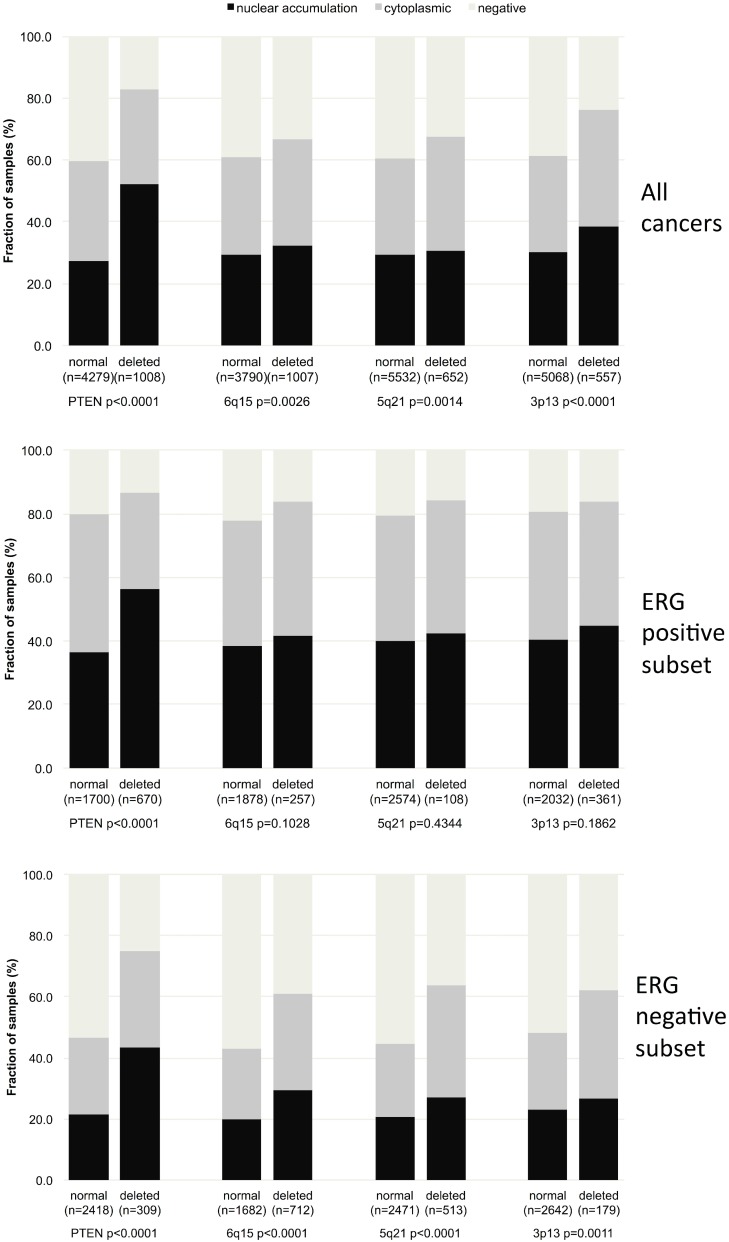
Association between GSK3ß localization and 10q23 (*PTEN*), 5q21 (*CHD1*), 6q15 (*MAP3K7*), 3p13 (*FOXP1*) deletion in all cancers, the ERG positive and the ERG negative subset

Comparison of GSK3ß expression with several of the most frequent genomic deletions in prostate cancer (*PTEN*, 3p13, 6q15 and 5q21) revealed that GSK3ß staining was strikingly linked to *PTEN* deletions (*p* < 0,0001, Figure 5). Weaker associations were also found with deletions of 6q15 (*p* = 0.0026), 5q21 (*p* = 0.0014) and 3p13 (*p* < 0.0001). However, subset analysis of ERG positive and ERG negative cancers revealed that the associations, with the exception of *PTEN,* were solely driven by ERG negative cancers (*p* ≤ 0.002 each).

### Association to tumor cell proliferation (Ki67LI)

Presence of GSK3ß staining was significantly linked to increased cell proliferation as measured by Ki67LI. This held true for purely cytoplasmatic but all the more for combined cytoplasmatic and nuclear staining (nuclear accumulation) (*p* < 0.0001; Table 2). These associations were independent from the Gleason grade as they also held true in subgroups of tumors with identical Gleason score (≤3+3, 3+4, 4+3 *p* < 0.0001 each and ≥4+4; *p* = 0.0101).

**Table 2:** Association between GSK3ß staining and Ki67 labeling index in all cancers and Gleason categories

Gleason	GSK3ß	N	Ki67-LI (Mean ± SEM)		P
All	Negative	2,416	2.0	0.05	<0.0001
	Cytoplasmic only	1,741	3.1	0.06	
	Nuclear accumulation	1,467	3.6	0.07	
≤3+3	Negative	678	1.8	0.08	<0.0001
	Cytoplasmic only	341	2.6	0.11	
	Nuclear accumulation	175	3.0	0.15	
3+4	Negative	1,297	2.0	0.06	<0.0001
	Cytoplasmic only	1,039	3.0	0.07	
	Nuclear accumulation	795	3.2	0.08	
3+4 Tertiary 5	Negative	94	2.3	0.24	<0.0001
	Cytoplasmic only	61	3.5	0.30	
	Nuclear accumulation	73	3.5	0.27	
4+3	Negative	169	2.1	0.23	<0.0001
	Cytoplasmic only	183	3.5	0.22	
	Nuclear accumulation	185	4.2	0.22	
4+3 Tertiary 5	Negative	91	2.2	0.39	<0.0001
	Cytoplasmic only	63	3.9	0.47	
	Nuclear accumulation	127	4.7	0.33	
≥4+4	Negative	86	3.5	0.52	0.0101
	Cytoplasmic only	53	4.8	0.67	
	Nuclear accumulation	109	5.5	0.47	

SEM, standard error of the mean.

### Association with PSA recurrence

**Figure 6:**
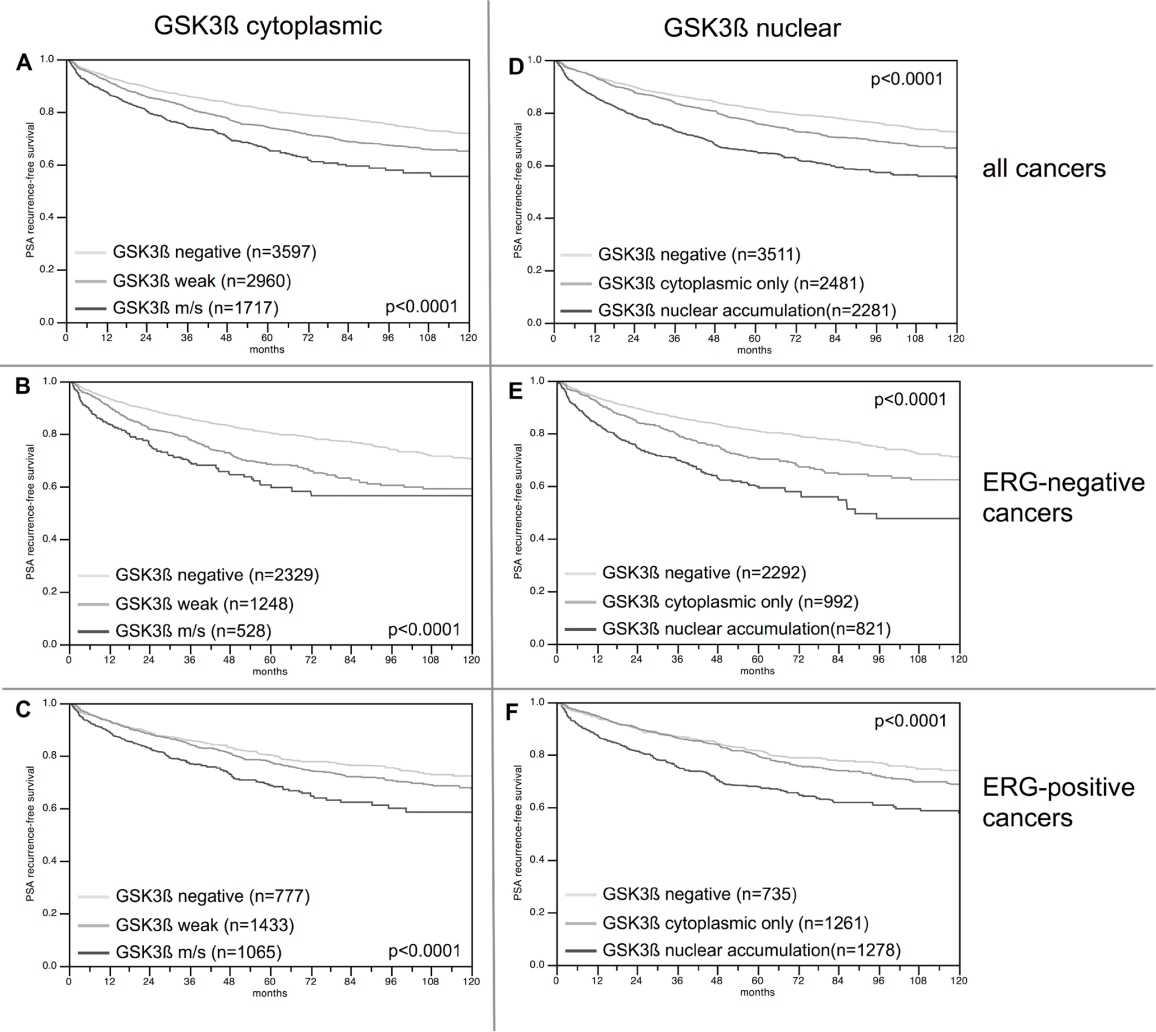
Kaplan–Meier analysis of PSA recurrence-free survival after radical prostatectomy and (**A**–**C**) cytoplasmic GSK3ß expression, and (**D**–**F**) nuclear GSK3ß accumulation in all cancers and the ERG negative and positive subset; m/s: moderate or strong cytoplasmic GSK3ß staining.

**Figure 7:**
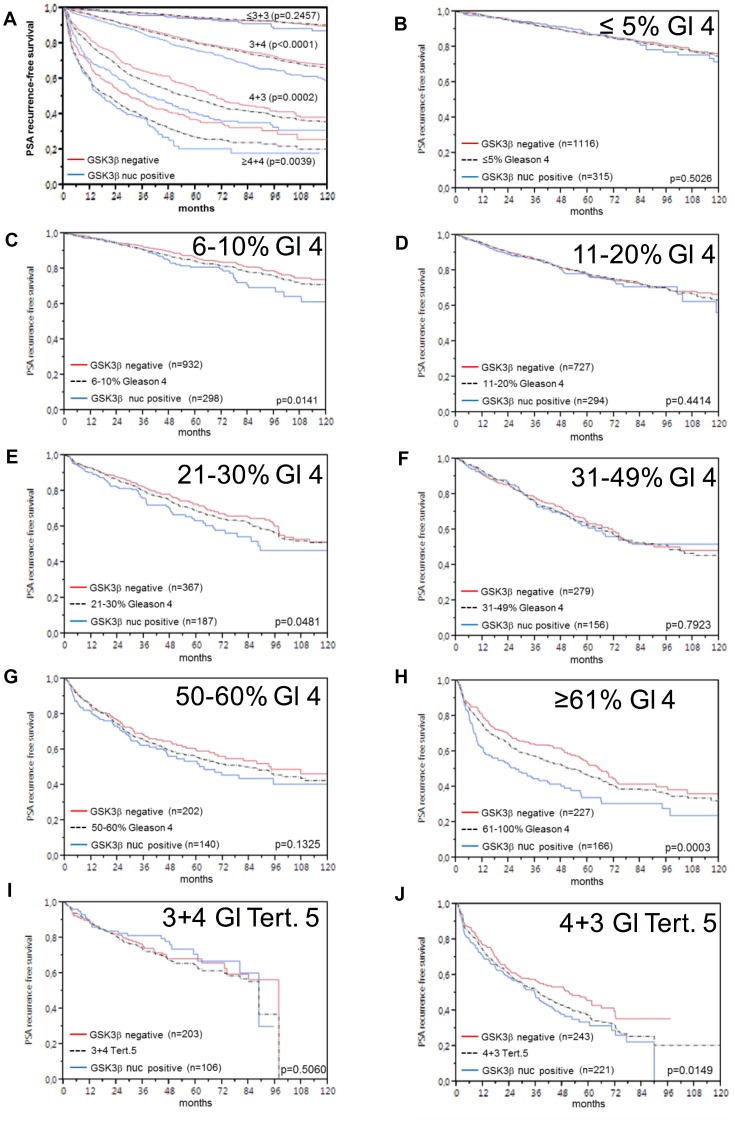
Prognostic impact of GSK3ß expression in subsets of cancers defined by the Gleason score (**A**) Impact of negative (red line) and nuclear positive (blue line) *GSK3ß* expression as compared to the classical Gleason score categories (indicated by black dotted lines). (**B**–**H**) Impact of negative (red line) and nuclear positive (blue line) *GSK3ß* expression in the quantitative Gleason score categories (black dotted lines) defined by the percentage of (**B**) ≤5%, (**C**) 6–10%, (**D**) 11–20%, (**E**) 21–30%, (**F**) 31–49 %, (**G**) 50–60%, (**H**) ≥61% Gleason 4 patterns. (**I**–**J**) Impact of Gleason score categories (**I**) 3+4 and (**J**) 4+3 with tertiary (tert.) Gleason 5 patterns.

Follow-up data were available from 8,598 patients with interpretable GSK3ß staining on the TMA. The intensity of cytoplasmic GSK3ß staining was strongly linked to early biochemical recurrence (*p* = 0.0001, Figure 6A). Factoring in the staining localization revealed that the prognosis of GSK3ß positive cancers deteriorated if the protein accumulated in the nucleus (*p* < 0.0001, Figure 6B). These findings were independent of the ERG status (Figure 6B, 6C and 6E, 6F). To better understand the prognostic impact of nuclear GSK3ß accumulation, we performed subset analyses in tumors with comparable classical and quantitative Gleason grades. These analyses revealed that nuclear GSK3ß expression measurement did provide additional prognostic impact in morphologically well-characterized tumors with Gleason 3+4 (*p* < 0.0001) and Gleason 4+3 (*p* = 0.0002, Figure 7A). Expansion of the subgroup analysis to the quantitative Gleason grade showed that nuclear GSK3ß accumulation even had a prognostic impact in several subsets of tumors with comparable fractions of Gleason 4 (Figure 7B–7H).

### Multivariate analysis

Four different models of multivariate analyses were evaluated (Table 3, Supplementary Table 3). Scenario 1 evaluated the postoperatively available parameters (pathological tumor stage, pathological lymph node status (pN), surgical margin status, preoperative PSA value and pathological Gleason grade obtained after the evaluation of the entire resected prostate and nuclear GSK3ß expression). In scenario 2 pN was excluded. This approach can markedly increase case numbers and power of the test. Two additional scenarios 3 and 4 model the preoperative situation as much as possible. Since postoperative determination of a tumor's Gleason grade is “better” than the preoperatively determined Gleason grade (subjected to sampling errors and consequently under-grading in more than one third of cases [24]), scenario 3 included the postoperative Gleason grade instead of the Gleason grade originally obtained at biopsy in scenario 4. Nuclear GSK3ß accumulation provided highly significant prognostic value beyond the established pre- and postoperative parameters in all scenarios irrespective of the ERG status (*p* < 0.0001 for all scenarios). The univariate hazard ratio of nuclear GSK3ß accumulation for PSA recurrence-free survival was a moderate 2.06 (95% CI 1.86-2.29, *p ≤* 0.0001). The multivariate hazard ratio varied from 1.72 to 1.37 depending on the model used (Supplementary Table 3).

**Table 3:** Multivariate analysis with established prognostic parameters and the GSK3ß localization in all cancers, the ERG negative and positive subset

Subset	Scenario	N	P							
			Preoperative PSA-Level	pT stage	cT stage	Gleason grade prostatectomy	Gleason grade biopsy	pN stage	R status	GSK3ß-localisation
**All cancers**	1	5,057	<0.0001	<0.0001	-	<0.0001	-	<0.0001	0.0026	<0.0001
	2	8,086	<0.0001	<0.0001	-	<0.0001	-	-	<0.0001	<0.0001
	3	7,987	<0.0001	-	<0.0001	<0.0001	-	-	-	<0.0001
	4	7,884	<0.0001	-	<0.0001	-	<0.0001	-	-	<0.0001
**ERG-negative**	1	2,575	0.0003	<0.0001	-	<0.0001	-	0.0131	0.1651	<0.0001
	2	4,020	<0.0001	<0.0001	-	<0.0001	-	-	0.0005	<0.0001
	3	3,988	<0.0001	-	<0.0001	<0.0001	-	-	-	<0.0001
	4	3,934	<0.0001	-	<0.0001	-	< 0.0001	-	-	<0.0001
**ERG-positive**	1	2,037	0.0001	<0.0001	-	<0.0001	-	0.0164	0.0075	<0.0001
	2	3,199	<0.0001	<0.0001	-	<0.0001	-	-	<0.0001	0.0001
	3	3,138	<0.0001	-	<0.0001	<0.0001	-	-	-	0.0002
	4	3,099	<0.0001	-	<0.0001	-	<0.0001	-	-	<0.0001

Scenario 1 includes all postoperatively available parameters (pathological tumor (pT) stage, lymph node status (pN), surgical margin (R) status, preoperative PSA value and Gleason grade obtained after the morphological evaluation of the entire resected prostate. Scenario 2 excluded the nodal status from analysis. Scenario 3 included preoperative PSA, clinical tumor (cT) stage and Gleason grade obtained on the prostatectomy specimen. In scenario 4, the preoperative Gleason grade obtained on the original biopsy was combined with preoperative PSA, and cT stage.

## DISCUSSION

The results of our study demonstrate that nuclear GSK3ß protein accumulation is a moderate and independent predictor of poor prognosis in prostate cancer.

Cytoplasmic GSK3ß staining - with or without additional nuclear staining - was seen in 57% of 9,164 interpretable prostate cancers, while normal prostatic epithelial tissue was negative under the selected experimental conditions. Our results fit well to earlier work. Li *et al*. described higher cytoplasmic GSK3ß expression in 499 cancers as compared to 491 normal prostate samples using a customized IHC score [15]. Darrington *et al*. found cytoplasmic and nuclear GSK3ß expression in 30% of 79 cancers but no GSK3ß staining in normal prostate epithelium [22]. The somewhat lower rate of GSK3ß positivity in the latter study as compared to our analysis has most likely technical reasons, including different antibodies (our study: Cell Signaling Technology #12456 1:900; Darrington *et al*.: New England Biolabs #27C10 1:20) and different IHC protocols (our study: autoclave pretreatment in pH7.8 TRIS-EDTA buffer, Darrington et al.: microwave pretreatment in pH6 citrate buffer). Others and we have demonstrated earlier that protocol modifications can dramatically impact the fraction of positive tissues in IHC experiments [25–28].

The most important finding of our study was a massive link between prostate cancer aggressiveness and translocation of the GSK3ß protein from the cytoplasm to the nucleus. In particular, nuclear GSK3ß accumulation was associated with adverse tumor features, including advanced pathological tumor stage, high Gleason grade, lymph node metastasis, elevated tumor cell proliferation and early PSA recurrence. It was not surprising to find the same associations (although weaker) for cytoplasmic staining, since nuclear accumulation was generally paralleled by a higher level of cytoplasmic GSK3ß staining. It is, thus, in line with our results that earlier studies focusing on cytoplasmic staining reported comparable associations with high Gleason score [22], advanced clinical stage, lymph node metastasis, extra-capsular extension, high Gleason score and an increased risk of biochemical recurrence [15]. However, the particular strong prognostic impact of nuclear staining suggests, that tumor relevant functions of GSK3ß exist which are specifically linked to its nuclear localization. This is supported by earlier work on the nuclear functions of GSK3ß. Several studies showed that GSK3ß forms complexes with various cancer-relevant proteins specifically in the nucleus, including cyclin D1 [29], STAT [30], GATA-4 [31], c-myc [32], NRF2 [33], Snail [34] and p53 [35]. Schütz et al [17] showed that inhibition of GSK3ß induces nuclear export of the AR in prostate cancer cells. Thus nuclear GSK3ß increases nuclear AR even in the absence of androgens supporting the growth of prostate cancer cells. Accordingly, data are accumulating which suggest a general role of nuclear GSK3ß accumulation for cancer aggressiveness. For example, a shift from cytoplasmic to nuclear expression also paralleled progression of pancreatic cancer [13]. Studies describing a relationship between GSK3ß overexpression and poor patient outcome in breast [6, 7], ovarian [8], oral cavity [9], urinary bladder [10], lung [11] and gastric cancer [12] also regularly found nuclear GSK3 localization to be decisive for prognosis.

To learn more about the molecular events associated to GSK3ß up-regulation in prostate cancer, we made use of the molecular database attached to our TMA. The *TMPRSS2:ERG* gene fusion occurs in 40–60% of prostate cancers, and results in deregulation of more than 1,600 genes [23, 36, 37]. Activation of Wnt signaling belongs to the best-known consequences of *ERG* fusion [36, 38, 39]. Wnt signaling stabilizes the transcription co-factor ß-catenin in the cytoplasm and triggers its translocation into the cell nucleus [40]. That GSK3ß controls Wnt signaling by inactivation of ß-catenin both in the cytoplasm [41, 42] and in the nucleus [43] might, thus, explain the predominance of nuclear GSK3ß in ERG positive cancers in our study. This assumption is further supported by earlier work showing that GSK3ß is up-regulated and translocated to the nucleus in response to activation of Wnt signaling [44, 45]. Other genes of interest with respect to GSK3ß include the *AR* and the *PTEN* tumor suppressor. The strong association between GSK3ß up-regulation and AR expression as well as *PTEN* loss in our study is in line with earlier work. For example, Mulholland *et al*. suggested a promiscuous growth signaling network governed by PTEN, AR, and GSK3ß, in which GSK3ß and *PTEN* loss cooperate for the progression to androgen-independent prostate cancer [19]. In this network, PTEN/GSK3ß signaling is believed to be at least partly functionally interchangeable with Wnt/ß-catenin signaling [19]. Moreover, GSK3ß has been shown to stabilize the AR protein and to enhance AR dependent transcription in some studies [46, 47], which fits well to the almost linear association between AR expression and both cytoplasmic and nuclear levels of GSK3ß in our study.

Besides deletions of PTEN, loss of certain small and large chromosomal regions is another hallmark of prostate cancer. Data from next generation sequencing studies demonstrate that such deletions are more prevalent than mutations of coding genes and many of these deletions have been linked to either ERG positive (i.e. PTEN and 3p13) or ERG negative cancers (i.e. 6q15 and 5q23) [48–52]. Finding a link between all of these deletions and GSK3ß up-regulation – exclusively in the subset of ERG negative cancers – suggests that GSK3ß might contribute to genomic instability at least in the absence of ERG. Several specific functions of GSK3ß and clinical observations are compatible with this assumption. GSK3ß is critically involved in microtubule remodeling [53], and it was shown to localize to the spindle pole in mitosis [54]. That many GSK3-inhibitors have been shown to cause chromosome misalignment and miss-segregation [55] strongly supports a functional link between disturbed GSK3ß homeostasis, failure of the spindle apparatus, and loss of genome integrity. We can only speculate why relevant associations between GSK3ß and genomic deletions were absented in ERG fusion positive cancers. However, it cannot be excluded that one or more target genes of ERG interfere with mechanisms linking GSK3ß to microtubule functionality. One example is the microtubule-associated protein Tau [56]. We have earlier shown that critical components of microtubules or their turnover, such as ßIII-tubulin [57] or Tau protein (Schroeder *et al*., submitted), are strongly up-regulated in ERG positive as compared to ERG negative cancers.

That GSK3ß analysis provided additional prognostic information beyond the established preoperative and postoperative prognostic parameters in prostate cancer makes it a promising candidate for potential routine diagnostic applications. Remarkably, the analysis of the prognostic role of nuclear GSK3ß up-regulation in subgroups of prostate cancers that were narrowly defined by identical classical and quantitative Gleason grades suggest a limitation of the prognostic value of GSK3ß measurement to cancers with Gleason grade 3+4 and 4+3. This limitation of the prognostic impact to these subgroups is not disappointing as these tumors are subject to the most difficult therapeutic decision making with options ranging from active surveillance to prostatectomy. The Gleason grading system is purely based on the simple distinction of architectural features, neglects any cytological criteria, but is statistically extremely powerful. The prognostic power of the Gleason grade is much higher than the histologic grading in various other cancer types, such as for example kidney cancer [58] or invasive bladder cancer [59]. This holds true if the Gleason grading method is limited to 5 prognostic subgroups [60]. Based on the analysis of a cohort of more than 10,000 prostate cancers available at our institution, we had earlier shown that Gleason Grade information can be refined by using the percentage of Gleason 4 grades as a continuous variable. Both in biopsies and in prostatectomy samples, prostate cancer prognosis deteriorates gradually with increasing percentage of Gleason 4 pattern (quantitative Gleason Grade) [61]. That nuclear accumulation of GSK3ß is even prognostically relevant in some prostate cancer subgroups defined by a comparable Gleason 4 fraction provides further arguments for a possible clinical application of GSK3ß analysis for assessing prostate cancer aggressiveness.

The therapeutic potential of GSK3ß inhibition has become an important area of investigation. More than 50 compounds targeting GSK3ß have been described as to yet [4]. Evidence for a possible therapeutic application of some of these drugs in cancer comes from *in-vitro* and *in-vivo* xenograft models. For example, GSK3ß inhibition reduces cell proliferation, increases apoptosis, and sensitizes to gemcitabine in pancreatic cancer cells [62], reduces viability of ovarian cancer cells [8], increases apoptosis in colon cancer cells [63], and reduces cell proliferation and survival in lung cancer cells [11]. Clinical phase I/II trials have been completed in patients with acute leukemia (NCT01214603) and advanced or metastatic solid cancers (NCT01287520) using the GSK3ß inhibitor LY2090314, and another phase II study on metastatic pancreatic cancer (NCT01632306) is currently recruiting participants. Should these studies provide evidence for a clinical benefit of GSK3ß inhibition in cancer therapy, the results of our study would justify evaluating prostate cancer in future GSK3ß inhibition trials.

In summary, the results of our study identify nuclear GSK3ß accumulation as a moderate and independent prognosticator in prostate cancer. GSK3ß expression analysis has the potential for a clinical routine application – either alone, or more likely, in combination with other biomarkers.

## MATERIALS AND METHODS

### Patients

Radical prostatectomy specimens were used from 12,427 patients, who had surgery between 1992 and 2012 (Department of Urology and the Martini Clinic at the University Medical Center Hamburg-Eppendorf). Specimens were analyzed by a standard procedure with embedding of the entire prostate for histological analysis [25]. In addition to the classical Gleason categories, “quantitative” Gleason grading was performed as described before [61]. Median follow-up was 48.9 months (range: 1 to 275 months; Table 4). PSA recurrence was defined as the time point when the postoperative PSA level was at least 0.2 ng/ml and increasing at subsequent measurements. Tissue microarrays (TMA) were produced as described earlier in detail [64]. Each TMA block contained various control tissues and normal prostate. The TMA was annotated with results on ERG expression, *ERG* break apart FISH analysis [65] and deletion status of 5q21 (*CHD1*) [48], 6q15 (*MAP3K7*) [48], *PTEN* (10q23) [49], 3p13 (*FOXP1*) [50], KI67 labeling Index (Ki67LI) [66] and androgen receptor (AR) expression [23]. The usage of archived diagnostic leftover tissues for TMAs and their analysis for research purposes has been approved by local laws (HmbKHG, §12a) and by the Ärztekammer Hamburg (WF-049/09). The work has been carried out in compliance with the Helsinki Declaration.

**Table 4:** Composition of the prognosis tissue microarray containing 12 427 prostate cancer specimens

	No. of patients	
	Study cohort on tissue microarray	Biochemical relapse among categories
**Follow-up**		
*n*	11 665	2 769 (23.7%)
Mean/Median (month)	56.3/48.9	
**Age (y)**		
≤50	334	81 (24.3%)
51–59	3 061	705 (23%)
60–69	7 188	1 610 (22.4%)
≥70	1 761	370 (21%)
**Pretreatment PSA (ng/ml)**		
<4	1 585	242 (15.3%)
4–10	7 480	1 355 (18.1%)
10–20	2 412	737 (30.6%)
>20	812	397 (48.9%)
**pT stage (AJCC 2002)**		
pT2	8 187	1 095 (13.4%)
pT3a	2,660	817 (30.7%)
pT3b	1 465	796 (54.3%)
pT4	63	51 (81%)
**Gleason grade**		
≤3+3	2 848	234 (8.2%)
3+4	6 679	1 240 (18.6%)
3+4 Tertiary 5	433	115 (26.6%)
4+3	1 210	576 (47.6%)
4+3 Tertiary 5	646	317 (49.1%)
≥4+4	596	348 (58.4%)
**pN stage**		
pN0	6 970	1 636 (23.5%)
pN+	693	393 (56.7%)
**Surgical margin**		
Negative	9 990	1 848 (18.5%)
Positive	2 211	853 (38.6%)

In the column “Study cohort on tissue microarray” numbers do not always add up to 12 427 in different categories because of cases with missing data. Percent in column “Biochemical relapse among categories” refers to the fraction of samples with biochemical relapse within each parameter in the different categories. Abbreviation: AJCC, American Joint Committee on Cancer.

### Immunohistochemistry (IHC)

Freshly cut TMA sections were immunostained on the same day and in one experiment. Slides were deparaffinized and exposed to antigen retrieval for 5 minutes at 121° C in pH 7.8 Tris-EDTA buffer. Primary antibody specific for total GSK3ß (rabbit monoclonal antibody, Cell Signaling Technology, USA; cat#12456; dilution 1:900) was applied at 37° C for 60 minutes. Bound antibody was then visualized using the EnVision Kit (Dako, Glostrup, Denmark) according to the manufacturer's directions. GSK3ß staining of variable intensity was seen in the cytoplasm, which was often accompanied by nuclear co-staining of similar intensity. Since GSK3ß positive cancers typically showed staining of all (100%) tumor cells, we recorded the cytoplasmic staining intensity (0 (negative), 1+(weak), 2+ (moderate), and 3+ (strong) as well as the presence or absence of nuclear co-staining for each tissue spot, but not the percentage of stained tumor cells.

### Statistics

Statistical calculations were done with JMP^®^ 10 (SAS Institute Inc., NC, USA). Contingency tables and chi^2^-test were performed to look for associations between molecular parameters and tumor phenotype. Kaplan–Meier survival curves were calculated and tested with the log-rank test for significant differences between groups. Cox proportional hazards regression analysis tested the statistical independence and significance between pathological, molecular and clinical variables in various clinical models.

## SUPPLEMENTARY MATERIALS FIGURES AND TABLES


