# Supplementary material for: A nuclear shift of GSK3β protein is an independent prognostic factor in prostate cancer
Source: Oncotarget. 2019 Mar 1;10(18):1729–44. doi: 10.18632/oncotarget.26739 (PMC6422199; doi:10.18632/oncotarget.26739)
Supplement: Supplementary file 1 [file oncotarget-10-1729-s001.pdf]

A nuclear shift of GSK3β protein is an independent prognostic factor in prostate cancer

SUPPLEMENTARY MATERIALS

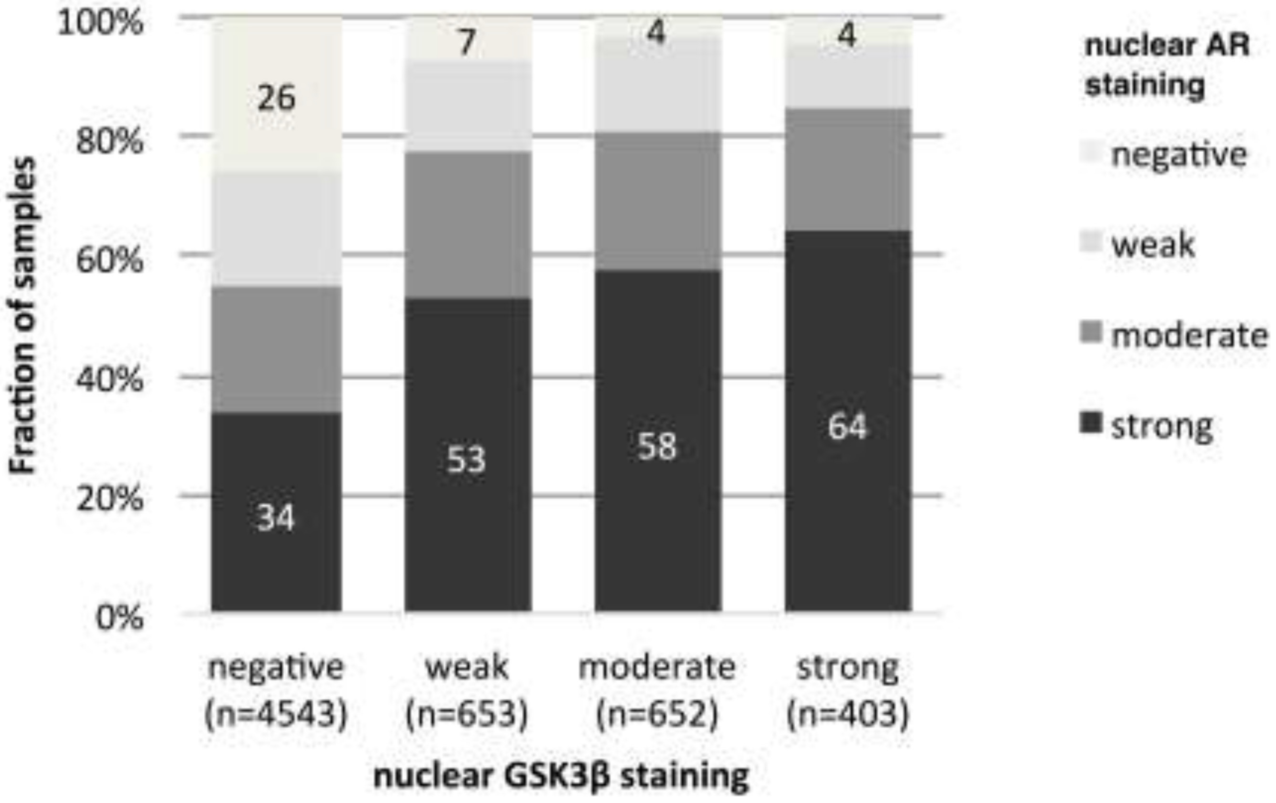

Supplementary Figure 1: Association between nuclear GSK3ß staining and expression of nuclear androgen receptor (AR)

Supplementary Table 1: Association between GSK3ß staining results and prostate cancer phenotype in *ERG* fusion negative tumors

		GSK3ß cytoplasmic (%)					GSK3ß cytoplasmic and nuclear (%)		
**Parameter**	***N***	**Negative**	**Weak**	**Moderate**	**Strong**	***P***	**Cytoplasmic only**	**Nuclear accumulation**	***P***
**ERG negative**	4,553	56.5	30.6	12.1	0.8		24.2	20.3	
**Tumor stage**									
pT2	3,039	60.3	28.9	10.2	0.6		23.9	16.6	
pT3a	936	52.7	33.4	13.4	0.5	<0.0001	23.7	24.5	<0.0001
pT3b-pT4	574	42.2	34.7	20.6	2.4		26.7	33.0	
**Gleason grade**									
≤3+3	889	74.4	20.6	5.0	0.1		18.0	8.2	
3+4	2,425	57.7	31.0	10.6	0.6		24.6	18.5	
3+4 Tertiary 5	206	48.5	36.4	14.6	0.5	<0.0001	24.8	27.2	<0.0001
4+3	490	39.4	41.0	18.0	1.6		33.3	29.0	
4+3 Tertiary 5	272	40.4	34.2	23.5	1.8		26.1	33.8	
≥4+4	268	39.9	32.1	25.0	3.0		22.0	40.7	
**Lymph node metastasis**									
N0	2,660	53.8	32.1	13.2	1.0	<0.0001	24.81	22.48	<0.0001
N+	257	38.1	34.6	24.1	3.1		29.18	35.02	
**Preoperative PSA level (ng/ml)**									
<4	570	56.8	28.3	14.0	0.9		26.1	17.4	
4-10	2,597	56.1	31.7	11.3	0.9	0.071	24.7	20.1	0.0016
10-20	994	58.8	29.5	11.3	0.5		23.0	19.1	
>20	372	53.5	28.8	16.7	1.1		19.6	28.5	
**Surgical margin**									
Negative	3,613	57.5	30.6	11.1	0.8	0.0033	24.2	19.1	0.0006
Positive	890	52.7	30.1	16.2	1.0		23.6	25.2	

Nuclear accumulation: nuclear staining with or without cytoplasmic co-staining.

Supplementary Table 2: Association between GSK3ß staining results and prostate cancer phenotype in *ERG* fusion positive tumors

		GSK3ß cytoplasmic (%)					GSK3ß cytoplasmic and nuclear (%)		
**Parameter**	***N***	**Negative**	**Weak**	**Moderate**	**Strong**	***P***	**Cytoplasmic only**	**Nuclear accumulation**	***P***
**ERG positive**	3,614	23.0	43.7	30.6	2.7		38.5	39.5	
**Tumor stage**									
pT2	2,139	24.1	45.3	28.4	2.2	0.0150	42.3	34.6	<0.0001
pT3a	1,009	20.1	42.9	33.4	3.6		36.7	44.3	
pT3b-pT4	464	23.9	38.5	34.8	2.8		26.5	52.6	
**Gleason grade**									
≤3+3	713	75.6	12.5	9.1	2.8	<0.0001	42.9	24.4	<0.0001
3+4	2,104	62.4	11.4	16.7	9.5		41.8	37.6	
3+4 Tertiary 5	111	51.4	14.4	15.3	18.9		35.1	48.7	
4+3	349	44.2	14.2	20.6	20.9		29.7	55.8	
4+3 Tertiary 5	193	31.1	14.5	25.4	29.0		20.7	68.9	
≥4+4	147	40.1	12.2	19.7	27.9		20.4	59.9	
**Lymph node metastasis**									
N0	2,115	58.7	12.1	16.6	12.6	<0.0001	39.0	41.3	<0.0001
N+	221	41.6	12.2	22.6	23.5		19.5	58.4	
**Preoperative PSA level (ng/ml)**									
<4	571	65.0	11.0	15.4	8.6	0.0775	42.4	35.0	<0.0001
4-10	2,134	61.2	11.7	16.0	11.1		39.4	38.8	
10-20	666	56.8	14.0	17.3	12.0		37.1	43.2	
>20	225	51.8	12.5	17.0	18.8		28.1	48.2	
**Surgical margin**									
Negative	2,820	62.2	12.3	15.5	10.0	0.0175	40.2	37.9	<0.0001
Positive	747	55.0	11.7	17.9	15.4		33.3	45.0	

Nuclear accumulation: nuclear staining with or without cytoplasmic co-staining.

Supplementary Table 3: Hazard ratios (95% confidence intervals) for biochemical relapse after prostatectomy for established risk factors and GSK3β localization in various scenarios

Model		Scenario 4	Scenario 3	Scenario 2	Scenario 1
**Variable**	**Analyzable (*N*)**	**8 081**	**8 207**	**8 309**	**8 295**
Gleason grade biopsy	≥4+4 *vs*. ≤3+3	3.90 (3.42–4.46)***			
cT stage	T2c *vs*. T1c	1.54 (1.40–1.69)***	1.46 (1.33–1.61)***		
Preoperative PSA level	≥20 *vs*. <4	3.75 (3.09–4.58)***	3.03 (2.50–3.69)***	2.08 (1.71–2.53)***	1.81 (1.49–2.21)***
GSK3β localization	Nuclear accumulation *vs*. negative	1.72 (1.55–1.92)***	1.53 (1.37–1.70)***	1.47 (1.32–1.63)**	1.46 (1.31–1.62)**
Gleason grade prostatectomy	≥4+4 *vs*. ≤3+3		11.4 (9.34–14.0)***	5.52 (4.45–6.85)***	4.51 (3.61–5.65)***
pT stage	T4 *vs*. T2			3.75 (2.56–5.32)***	3.29 (2.23–4.67)***
Surgical margin status	R1 *vs*. R0			1.47 (1.33–1.62)***	1.47 (1.33–1.62)***

Scenario 4 combines preoperatively available parameter (preoperative Gleason grade obtained on the original biopsy, clinical tumor (cT) stage, and preoperative PSA) with the postoperative GSK3β localization at the negative, low (weak and moderate) and strong intensity levels. In scenario 3 the biopsy Gleason is replaced by the Gleason grade obtained on radical prostatectomy (RPE). In scenario 2, cT-stage is superseded by pathological tumor (pT) stage and surgical margin (R) status. In scenario 1 the lymph node (pN) stage is added. Asterisk indicate significance level: $^{*}p \leq 0.05$, $^{**}p \leq 0.001$, and $^{***}p \leq 0.0001$.
